# Psychotherapists’ Trust, Distrust, and Generative AI Practices in Psychotherapy: Qualitative Study

**DOI:** 10.2196/88932

**Published:** 2026-04-02

**Authors:** Jinyan Kuang, Amber L Pope, Yixuan Zhang

**Affiliations:** 1 Department of Computer Science, William & Mary, 251 Jamestown Road, Williamsburg, VA, United States, 1 7572214842; 2 Department of School Psychology and Counselor Education, William & Mary, Williamsburg, VA, United States

**Keywords:** artificial intelligence, AI, generative AI, mental health, trust, qualitative methods

## Abstract

**Background:**

Generative artificial intelligence (GenAI) is increasingly used in mental health care, from client-facing chatbots to clinician-facing documentation aids. Psychotherapists’ willingness to rely on—or withhold reliance from—these tools has significant implications for care quality, yet little is known about how practicing clinicians calibrate trust and distrust in GenAI across tasks and contexts. Given that the therapeutic relationship is central to psychotherapy outcomes, understanding how GenAI intersects with this relational foundation is essential for responsible integration.

**Objective:**

This study aims to examine (1) psychotherapists’ experiences with, perceptions of, and trust or distrust in GenAI in therapeutic contexts and (2) how they perceive the role of GenAI within the therapeutic relationship and how their perceptions shape their trust and distrust in GenAI.

**Methods:**

We conducted a qualitative interview study using semistructured interviews with 18 actively practicing psychotherapists in the United States between January and May 2025. Participants were recruited through professional mailing lists, social media, and snowball sampling. Interviews (≈60 min each) were conducted via Zoom and explored psychotherapists’ experiences with, perceptions of, and trust or distrust in GenAI in therapeutic contexts. Data were analyzed using the general inductive approach, with iterative coding and team-based interpretation to identify themes.

**Results:**

Our findings show that psychotherapists’ GenAI adoption was highly individualized and contingent on maintaining professional role integrity—not merely technical oversight. Trust was sustained when GenAI operated in clinician-supervised, supportive roles for low-stakes tasks (eg, documentation and brainstorming), but diminished when control shifted, tasks involved high-stakes clinical judgment, or GenAI threatened to encroach on the authentic human connection central to therapy. Participants articulated conditions for trust that went beyond “human-in-the-loop” monitoring to include preservation of interpretive authority, ethical responsibility, and relational primacy. Distrust also extended to the broader sociotechnical ecosystem, including concerns about commercial incentives, insurance pressures, and the absence of clear organizational guidelines.

**Conclusions:**

Psychotherapists’ perspectives offer critical insights into GenAI’s current usages in their professional practices and the conditions under which they are willing to trust and distrust GenAI tools. Their experiences highlight the importance of maintaining clinician control, ensuring contextual appropriateness, and preserving the human connection central to psychotherapy. Future work should further examine how therapeutic orientation, professional experience, and client characteristics shape trust and distrust in GenAI. As GenAI becomes more embedded in mental health care, research is also needed to explore how specific GenAI system features can be responsibly designed to support clinical workflows and enhance therapeutic relationships. Organizational and policy frameworks will be essential to ensure responsible, ethically aligned, and human-centered GenAI deployment in psychotherapy.

## Introduction

The rapid advancement of generative artificial intelligence (GenAI) in mental health care has sparked both enthusiasm and concern in recent years [1-4]. Earlier mental health artificial intelligence (AI) tools were largely rule-based or template-driven, offering structured guidance [5]. With recent advancements in large language models, professional organizations have begun to respond. For example, the American Psychological Association (APA) is developing guidance to help psychologists integrate AI tools into practice in ways that respect ethical and legal obligations [6]. Yet a recent survey found that 71% of mental health practitioners had not incorporated AI-enabled tools into routine care [7]. Public and social-media discourse often portrays psychotherapists as broadly resistant to or distrustful of GenAI—casting the technology as incompatible with therapeutic work or as a deceptive substitute for human care, especially when chatbots impersonate psychotherapists [8]. These portrayals, however, say little about how practicing psychotherapists themselves are actively calibrating trust and distrust in GenAI in day-to-day clinical contexts.

Empirical work has begun to examine mental health professionals’ attitudes toward AI and GenAI. Survey and interview studies have shown mental health practitioners’ perceptions of AI in services [9-11], including views on the public’s use of AI chatbots for mental health support [12]. This body of work has identified perceived benefits for self-management and accessibility alongside concerns about data privacy, clinical reliability, and limited empathy. Complementary design research has explored early GenAI-augmented prototypes (eg, counselor agents and multimodal assistants) to probe possible roles for GenAI in assessment, client participation, and skill practice [13]. Together, existing work illuminates general attitudes and speculative futures, but it offers less insight into how psychotherapists currently use GenAI in practice, which concrete tasks they deem trustable or untrustable, and how they respond when clients independently bring GenAI into therapy.

Research on trust in AI provides a useful lens for these questions. Trust in AI is often defined as a belief that a system is reliable, competent, transparent, and responsive in a given context [14], whereas distrust reflects expectations of harm, manipulation, or misuse [15]. A large body of work shows that trust in automation is situational and conditional: people are more willing to rely on AI when conditions around evidence, oversight, and control are satisfied, and they withdraw trust when those conditions are violated [16-21]. Trust is thus not a fixed trait but a dynamic, task- and role-dependent judgment that evolves with experience. In high-stakes domains, miscalibrated trust—either overtrust that leads to uncritical reliance or undertrust that prevents appropriate use—can produce safety risks and missed opportunities [22].

Decades of research have established that the therapeutic relationship—often termed the “therapeutic alliance” or “working alliance”—is among the most important predictors of psychotherapy outcomes, frequently accounting for more variance than specific techniques or theoretical orientations [23]. The alliance encompasses agreement on therapeutic goals, consensus on tasks, and the affective bond between therapist and client [24]. Such a relational foundation makes psychotherapy a domain where trust is not merely a technical consideration but a fundamentally interpersonal one. Psychotherapy is thus inherently relational and context-sensitive [25,26], making questions of calibrated trust and distrust in GenAI especially salient. Misplaced trust could, for example, lead psychotherapists to overdelegate clinical reasoning, accept flawed recommendations, or input sensitive client data into insecure systems, undermining both care and confidentiality [27,28]. Insufficient trust, by contrast, may keep psychotherapists from using GenAI in low-risk areas where it could meaningfully augment care (eg, psychoeducation, homework support, or documentation) [29]. At the same time, decades of work on the therapeutic relationship underscores that the alliance between psychotherapist and client—the goals, tasks, and emotional bond of therapy—is central to outcomes [30,31]. Experiences with telehealth show that this relationship can adapt to new media but requires careful management of shifts in framing and presence [30]. As GenAI enters psychotherapy, the traditional dyadic relationship becomes a triad involving psychotherapist, client, and GenAI [32], raising open questions about GenAI’s appropriate role, boundaries, and influence on trust among all 3 parties.

Despite growing attention to GenAI in mental health, we know relatively little about how practicing psychotherapists form, calibrate, and revise trust and distrust in GenAI across tasks and contexts, or how they understand GenAI’s place in the evolving psychotherapist-client-GenAI relationship. In this work, we conducted a qualitative study with 18 psychotherapists to explore (1) What are psychotherapists’ experiences with, perceptions of, and trust or distrust in GenAI in therapeutic contexts? and (2) How do psychotherapists perceive the role of GenAI within the therapeutic relationship, and how do these perceptions shape their trust and distrust in GenAI?

## Methods

### Study Design

This study used a qualitative interview method to explore how psychotherapists perceive and use GenAI tools in their professional practice, with particular attention to the dynamics of trust and distrust. A qualitative approach was selected because it enables an in-depth understanding of psychotherapists’ lived experiences and contextualized decision-making around GenAI integration [33]. To do so, we conducted semistructured interviews via Zoom (Zoom Video Communications, Inc) between January and May 2025 and qualitative data analysis between May and July 2025 to further examine psychotherapists’ practices, trust (or distrust) of GenAI, and their envisioned relationship between GenAI and psychotherapists.

### Recruitment

Concerning the inclusion criteria*,* participants were eligible if they (1) held a valid license or credential to practice psychotherapy in the United States (eg, licensed professional counselor, licensed clinical social worker, and licensed psychologist), and (2) were actively providing psychotherapy to clients at the time of enrollment. No exclusion criteria were applied regarding therapeutic orientation, practice setting, years of experience, or prior familiarity with GenAI; we sought a diverse range of perspectives rather than a homogeneous sample.

Concerning participant recruitment, they were recruited through purposive and snowball sampling between January and May 2025. Recruitment advertisements were distributed via (1) professional mailing lists for psychotherapists and mental health practitioners, (2) LinkedIn posts targeting mental health professionals, and (3) professional networks of the research team. The recruitment message briefly described this study’s purpose (exploring psychotherapists’ perspectives on GenAI), eligibility criteria, time commitment (≈60 min), and compensation. Interested individuals contacted the research team via email and were screened for eligibility. We received 20 expressions of interest; 18 completed interviews, and 2 did not attend their scheduled sessions (no follow-up information was available regarding reasons for nonattendance).

### Interview Study Procedures

The semistructured interview guide was developed iteratively by the research team, drawing on (1) the existing literature on trust in AI and automation [21,22,34], (2) prior qualitative work on mental health practitioners’ attitudes toward AI [9,12], and (3) the research team’s clinical and human-computer interaction expertise. The guide was pilot-tested with 2 psychotherapists (not included in the final sample) to assess question clarity and flow, and minor revisions were made based on their feedback. The final guide included open-ended questions organized around three domains: (1) current experiences with GenAI in clinical practice, (2) perceptions of trust and distrust in GenAI across tasks and contexts, and (3) views on GenAI’s role in the therapeutic relationship. See Multimedia Appendix 1 for the complete interview protocol.

Before each interview, we asked each participant about their experience with GenAI and demographic information. If participants indicated previous GenAI experience, they were then prompted to elaborate on their experiences with GenAI use, followed by questions about their attitudes and trust or distrust of GenAI. When a participant had no prior GenAI experience, we first shared a web link from the APA [35] that outlines the potential clinical uses of GenAI and the associated ethical guidelines. The discussion then focused on the participants’ reactions and their anticipated levels of trust or distrust. In each interview, we also asked about views on the independent use of GenAI tools by clients and their perceptions of the relationship among the psychotherapist, GenAI, and client. The interview sessions lasted approximately 60 minutes. See Multimedia Appendix 1 for the detailed interview questions.

### Data Analysis

All interview sessions were transcribed verbatim and checked for accuracy against the original recordings. We conducted a qualitative analysis with the transcripts guided by the general inductive approach [36]. First, the lead author carefully read the transcripts multiple times to become familiar with the data and the whole brief analytic memos to capture initial impressions about psychotherapists’ GenAI practices, perceived benefits and risks, and expressions of trust and distrust of GenAI in different clinical contexts.

In the first cycle of coding, the lead author conducted line-by-line coding, assigning low-level, descriptive codes to segments of text that captured (1) concrete uses of GenAI in practice including both psychotherapists’ practices (eg, documentation, homework support, and supervision) and their observations of their clients’ usages (eg, journaling and emotional support), (2) conditions under which psychotherapists felt trust or distrust in using GenAI, and (3) how they perceived GenAI’s role in the psychotherapist-client relationship. Codes were kept close to participants’ language and iteratively refined as additional transcripts were reviewed. A provisional code list was updated after every 2‐3 transcripts to merge redundant codes, clarify labels, and add new codes that reflect concepts not captured previously.

Next, low-level codes were grouped into more abstract categories that summarized patterned meanings across participants and connected related codes (eg, “emergence of trust in GenAI for low-stakes administrative tasks,” “determining trust or distrust based on boundaries around high-stakes clinical reasoning,” “enhancing trust in guided client use of GenAI between sessions,” and “distrust about data privacy and commercialization”). Through constant comparison within and across transcripts, these categories were iteratively collapsed and reorganized into higher-level themes that described (1) psychotherapists’ current GenAI engagement, (2) how professional autonomy and task type set boundaries for trust or distrust, and (3) how GenAI is positioned within the therapeutic relationship, which aligns with their trust or distrust.

Throughout the coding process, 2 additional members of the research team independently coded overlapping subsets of transcripts using the evolving codebook and then met with the lead author in weekly analytic meetings to compare interpretations. Discrepancies in coding were discussed and resolved through group discussion until consensus was reached, and the shared codebook was iteratively updated to reflect agreed-upon definitions and illustrative coding examples. As analysis progressed, the team regularly compared new transcripts against the existing codebook to ensure consistent application of codes while remaining open to emergent concepts. Data collection and analysis proceeded concurrently and continued until thematic saturation was reached, defined as the point at which no substantively new codes or themes emerged in successive interviews and additional transcripts primarily confirmed the existing thematic structure. This study adhered to the COREQ (Consolidated Criteria for Reporting Qualitative Research) checklist [37]. See Checklist 1 for the detailed checklist.

### Ethical Considerations

Ethical approval for this study was obtained from the Institutional Review Board at William & Mary (PHSC-2024-06-30-17113). Before participation, the research team explained this study’s purpose, procedures, potential risks, and benefits to all participants. Written informed consent was obtained via participants’ digital signatures before each interview. Each participant was assigned a unique study ID upon enrollment, and all identifying information was removed to ensure confidentiality. At the end of this study, each participant received a US $20 gift card as compensation upon completing the interview.

### Data Availability

The qualitative data (interview transcripts) generated during this study are not publicly available due to privacy and confidentiality concerns; transcripts contain sensitive information that could identify participants despite deidentification efforts. The interview guide is provided in Multimedia Appendix 1. Requests for access to deidentified data excerpts or the codebook may be directed to the corresponding author and will be considered on a case-by-case basis, subject to institutional review board approval and establishment of a data use agreement.

## Results

### Overview

Psychotherapists in our interview study (see a summary of participants’ demographics information in Table 1) reported experimenting with GenAI mainly in supporting, nonclient-facing tasks, using it as an assistant rather than a psychotherapist substitute (see a summary of usage contexts in Table 2). Psychotherapists described using GenAI in several practical ways. Some used GenAI to draft session notes from generic prompts (eg, “therapy note, client struggling in marriage*”*). Others explored GenAI-driven homework support to assist clients between sessions or used GenAI as a communication bridge when clients’ primary language differed from their own, allowing bilingual or multilingual clients to better articulate their thoughts and improving access for those who cannot easily find language-matched providers. Psychotherapists also applied GenAI for psychoeducation, using it to generate “simulated client” scenarios and offering more realistic and diverse practice opportunities than classmates. Participants valued GenAI’s potential to enhance efficiency, extend therapeutic reach, and diversify resources—yet they consistently paired these ideas with caveats about trust, control, and ethical boundaries, setting the stage for the next section.

**Table 1.** Overview of participants’ demographics information.

Dimension and response options	Interview participants (n=18), n
Working experiences^a^ (years) [38]	
1‐10	14
11‐20	2
21‐30	1
31 or more	1
Gender	
Woman	14
Man	4
Nonbinary	N/A^b^
Race or ethnicity	
Asian	2
Black or African American	3
White	13
Prefer to self-describe	1

a Mean 9.7, SD 8.5 years; median 7.5, IQR 13 years.

b N/A: not applicable.

**Table 2.** Contexts and example quotes of GenAI^a^ use among psychotherapists.

Usage contexts	PIDs^b^	Example quotes of participants’ GenAI use
Administrative and clinical support		
Clinical documentation	P02, P04, P07, P11	“I get [clients’] consent and then it records the session; from that transcript it will generate my note, and I edit that note and put it into my EHR.”
Translation and language	P01, P05, P16	“I had ChatGPT translate a breathing exercise into Spanish for one of my clients.”
Treatment plan formulation	P11, P14, P17	“I’ve used GenAI the most for wording a treatment plan goal.”
Personalized homework creation	P01, P02, P03, P05, P09, P13	“I asked ChatGPT to make me a worksheet [that contains] information from books that I think provide a good model and explain it in layman’s terms versus clinical terms and ask ChatGPT to make reflection questions for my client.”
Training and supervision		
Ideation and brainstorming	P03, P06, P13, P18	“I need kind of a brainstorming colleague thing that I have [Gen]AI help me create content, like I might say, please give me 3 paragraphs of a case conceptualization for a client who’s experiencing 3 anxiety symptoms.”
Roleplay scenarios and scripts	P01, P11, P15	“Give me a real textbook kind of description of a persona that I can use in my training materials.”
Supervision and clinical consultation	P09	“I’ve even considered if I could run a case by a [Gen]AI to get a sort of second opinion, almost like an ’AI supervisor’ giving me feedback.”
Self-help and emotional support	P06, P11, P12	“I kind of used a GenAI [chatbot] for me personally, when I was going through a difficult time where I interacted with the GenAI to get support.”

a GenAI: generative artificial intelligence.

b PID: participant ID.

Based on their practices in GenAI in therapeutic contexts, we further identified 2 key domains that shape their trust and distrust toward GenAI in therapeutic contexts. First, psychotherapists articulated conditional trust: trust in GenAI that was bounded by professional autonomy, task specificity, and careful oversight. Second, they reflected on GenAI’s position within the therapeutic relationship, weighing scenarios where it might enhance client engagement against fears it could erode the authentic human bonds central to psychotherapy.

### Theme 1: Professional Autonomy Sets Boundaries for Trust

#### Overview

Theme 1 addresses psychotherapists’ trust in GenAI for their own professional use—that is, when they themselves use GenAI tools in clinical workflows. Our participants conveyed that their trust in GenAI was conditional*—*bounded by a sense of self-regulation, task-specific usage, and personal limits. For psychotherapists, the relevant conditions revolve around safeguarding the therapeutic frame, protecting client confidentiality, and preserving the clinician’s professional agency. In practice, psychotherapists’ trust in GenAI remains high only when they can maintain direct control over how the technology is used. When that control slips, such as using GenAI for unfamiliar tasks or allowing others to dictate its use, their trust quickly diminishes.

#### Subtheme 1: “Hand on the Wheel” Rule

Participants highlighted that their trust in GenAI systems did not stem from the technology itself, but from their perceived ability to manage and control [Gen]AI’s involvement. Psychotherapists in our study articulated a personal “hand on the wheel” “boundary” for trust*:* trust arose primarily when they could directly influence the GenAI’s actions through careful prompting, reviewing outputs, and maintaining options to delete or redact any generated content. In other words, psychotherapists’ trust was grounded less in confidence in GenAI itself than in confidence in their own professional ability to control the system: their judgment, expertise, and responsibility. For example, 1 psychotherapist (P03) tied her trust to her ability to steer the GenAI with well-crafted prompts and usage limits:

*I trust myself to be a good prompter... I know my boundaries and I know what I’m comfortable doing and not doing. And so as long as I stick within my boundaries, I feel pretty comfortable using [Gen]AI, kind of accomplishing the goals that I have.*

[P03]

P03’s quote illustrates that trust is contingent not just on the quality or performance of the GenAI system, but rather on the clinician’s professional autonomy and self-assessment of their capability to manage interactions with GenAI appropriately. Crucially, this sense of control applied not only to what information she fed into the system, but also over the fate of the outputs: where the text was stored, how long it persisted, and whether it could be edited or deleted to preserve client confidentiality.

Another participant (P04) emphasized that trust was fragile and tied directly to control over sensitive clinical data. Describing the workflow of a GenAI note-taking tool, P04 explained that their trust hinged entirely upon the continued availability of features, such as data deletion and transcript management:

*With Blueprint, I can delete the transcript, I can wipe it off of that system and that is within my control... and so that makes me trust it and I would be distrustful if they took that away... like that’s the premium feature is to delete your transcript.*

[P04]

P04’s explanation suggests how removing or monetizing such control-related features would undermine his trust. His confidence in the tool thus rests on specific capabilities allowing direct oversight and editability. When those capabilities are guaranteed, he feels safe using the GenAI; if they are taken away, his trust would evaporate.

#### Subtheme 2: Task-Specific Boundaries of GenAI Trust

Beyond trusting their own abilities and control, psychotherapists’ trust in GenAI is also highly task-dependent. Participants generally viewed GenAI as suitable for low-stakes tasks, such as brainstorming intervention ideas or drafting preliminary homework assignments, because mistakes in these contexts carried minimal risk and could easily be corrected by the psychotherapist. Conversely, their trust diminished when GenAI attempted high-stakes or core clinical tasks, such as generating official clinical documentation or nuanced clinical reasoning. Psychotherapists are skeptical that GenAI can handle sensitive clinical decisions or documentation independently without error. P06 articulated this conditional trust, expressing comfort using GenAI for brainstorming but strong reservations about automated note-taking:

*I’m just not sure that I want a system recording a session and taking a note for me, because I don’t fully understand how that information is being gathered and stored, and I wouldn’t want my client’s information in a system that I didn’t fully understand.*

[P06]

Here, P06’s skepticism arose from uncertainty over how the GenAI processed, recorded, and stored sensitive session data. Allowing a GenAI to record therapy sessions and generate notes raises serious privacy and data security concerns. Even if the GenAI tools could technically produce a decent therapy note, the lack of transparency about data handling was enough to erode her trust. Similarly, P03 refused to use GenAI for core clinical reasoning about a client:

*I don’t ask ChatGPT to conceptualize my clients when it comes to trying to understand what’s wrong or what’s going wrong; I rely on people for that.*

[P03]

In psychotherapy, “conceptualization” refers to the process by which psychotherapists develop a comprehensive understanding of a client’s presenting issues, symptoms, underlying causes, and contextual factors [39]. It involves synthesizing information from assessments, clinical observations, and theoretical frameworks to inform diagnosis, treatment planning, and interventions. P03’s quote highlights that psychotherapists reserve essential tasks, such as case conceptualization, for their clinical judgment or consultation with human colleagues. P03 trusted GenAI as a tool to improve efficiency in peripheral tasks, but that trust stopped when the technology would encroach on areas requiring deep expertise, intuition, or accountability. In her view, having GenAI substitute for a psychotherapist’s interpretive or diagnostic role was beyond acceptable bounds—it challenged the clinician’s professional role and could compromise care.

In short, psychotherapists calibrate their trust in GenAI to the stakes of the task: routine or creative adjunct tasks are within bounds, whereas high-stakes therapeutic decisions are off-limits to GenAI.

#### Subtheme 3: Conditional Trust in Others' Use of GenAI

Another important boundary on psychotherapists’ trust emerges when considering other people’s use of GenAI. Our participants trusted themselves to use GenAI in a disciplined, responsible manner, but they withheld that trust from clients and even from less-experienced colleagues. Such disparity is rooted in psychotherapists’ confidence in their training, ethical standards, and understanding of GenAI’s limitations. That confidence drops when they imagine someone without similar expertise using GenAI in the therapy context. As P06 noted, even her high personal trust “probably drops if clients start to use [Gen]AI more and it impedes or interferes with the therapy.” Psychotherapists worried that others might not show the same caution or insight, raising the specter of misuse or overreliance on GenAI. P13 illustrated this concern by contrasting her own careful approach with the unpredictability of a client’s approach:

*I feel like because I’ve done my due diligence, because I know I have a technology background and know how to keep my own information confidential, I feel confident, using the [Gen]AI, the ways that I do. Personally, however, I don’t have that confidence that a client would be careful of what they share with a [Gen]AI.*

[P13]

P13’s concern suggests that psychotherapists feel they could control the flow of sensitive information when they themselves use GenAI, but their trust falters when that control shifts to clients. A client might unwittingly share private details with a GenAI without fully understanding the underlying risks, potentially breaching confidentiality. In the absence of the psychotherapist’s oversight, information security cannot be guaranteed, driving psychotherapists’ distrust of clients’ unsupervised GenAI use.

Psychotherapists also fear that both clients and junior clinicians could become overreliant on GenAI in ways that harm therapy or professional development. Half of our participants worried that clients might overimmerse themselves in GenAI-generated advice or content, losing contact with reality or bypassing the therapeutic process. Similar concerns apply to junior psychotherapists regarding excessive reliance on GenAI for clinical tasks (eg, note-taking and treatment plans), thereby missing out on learning critical skills and clinical reasoning. P04, an educator with positive personal experiences with GenAI-assisted note-taking, articulated concerns about the trainees:

*As an educator, I do have concerns...students still in school or early psychotherapists or counselors early in their training, early in their residency, not knowing how to create the notes.*

[P04]

P04 emphasized the importance of trainees developing foundational skills without excessive GenAI assistance. If beginners shortcut core tasks such as writing progress notes by relying on GenAI, they risk eroding their own clinical agency and expertise. Overreliance on GenAI at early career stages could produce future clinicians who lack essential skills in documentation, observation, and critical thinking.

Despite these concerns about others’ use of GenAI, psychotherapists did identify certain conditions that could increase their trust when clients or junior colleagues use GenAI. They did not categorically oppose others using GenAI; rather, they advocated for supervision and education to ensure safe and appropriate use. For clients, many psychotherapists suggested integrating a form of guided GenAI usage into therapy itself, essentially treating a client’s GenAI use as something to be discussed and managed within sessions. For example, P05 likened it to how psychotherapists already addressed clients’ social media habits:

*(It’s) helping clients navigate the use of [Gen]AI for positive means…just like we have to help clients navigate the use of social media in a positive way. That could be a part of the conversation—an intake: Do you use Snapchat? How often do you use [Gen]AI? What do you use [Gen]AI for? Do you use it to shop? Do you use it to write papers?*

[P05]

P05 provided a practical framework for supervising client GenAI use: openly ask about and discuss it during therapy (even as early as the intake interview), just as one would assess other aspects of a client’s digital life. Such a proactive approach could help clients use GenAI in a healthy, privacy-conscious manner. It also gives the psychotherapist insight and oversight, which in turn bolsters their trust in how the client is engaging with GenAI outside of sessions. That said, participants acknowledged that not all clients required the same level of monitoring. P12 pointed out that some client populations—she gave the example of first responders—come in with high digital security awareness and a critical stance toward technology. These clients might actually self-regulate their GenAI use responsibly, sometimes even outpacing the psychotherapist’s knowledge of the tool:

*I trust my first-responder clients. They research things on their own, know how to ask questions safely, and decide for themselves whether to rely on a [Gen]AI suggestion. Honestly, by the time they get to session, they sometimes know more about the tool’s data flow than I do.*

[P12]

P12’s experience highlights how individual client characteristics, such as professional background, technology literacy, and skepticism, can increase a psychotherapist’s trust in that client’s independent GenAI use. This contrasts with other clients, indicating that psychotherapists’ trust in others’ GenAI use is not one-size-fits-all; it must be calibrated to the person’s abilities and context.

Overall, psychotherapists remain cautious about integrating GenAI into clinical work, even as they acknowledge potential benefits in efficiency and support. Lingering concerns about risks keep their distrust ever-present in the background. In short, we found that psychotherapists’ trust in GenAI is highly conditional—closely tied to maintaining personal control, selecting appropriate tasks, and ensuring proper contexts for GenAI use. These conditions matter because trust is inseparable from psychotherapists’ professional role: safeguarding clinical judgment, ethical responsibility, and accountability for client outcomes [40] makes the question of what, how, and by whom GenAI is used central to their identity as psychotherapists.

### Theme 2: Trust, Distrust, and GenAI in Therapeutic Relationships

#### Overview

Theme 2 shifts focus to the relational context, examining trust and distrust as they arise when clients use GenAI independently, when GenAI is positioned within the therapeutic relationship, and when systemic pressures shape the perceived role of GenAI in therapy. Psychotherapists in our study further negotiated their trust in GenAI against potential distrust, largely depending on how GenAI was positioned within the therapeutic relationship. On one hand, their observations of clients independently using GenAI for therapeutic tasks bolstered initial trust and confidence in GenAI tools. On the other hand, that trust became fragile and often flipped to skepticism when psychotherapists imagined GenAI encroaching on the human bonds that underlie effective therapy. These tensions shaped how psychotherapists envisioned GenAI’s future role as either aligning with their professional values or conflicting with them.

#### Subtheme 1: Psychotherapists’ Attitudes in Client-Led GenAI Use as a Complement to the Therapeutic Alliance

Many psychotherapists expressed cautious trust in GenAI based on seeing their clients incorporate tools such as ChatGPT into therapy-related activities in situations where their clients already engaged in active therapy, and psychotherapists could integrate and guide GenAI use within the therapeutic frame. Clients had used GenAI for tasks such as journaling and homework (P01), conversational practice or advice (P06), and organizing thoughts or self-diagnosing symptoms (P13). Psychotherapists generally viewed these independent uses of GenAI by clients as beneficial and not as a threat to their own role. Instead, they described GenAI as a complementary tool that could enhance openness, confidence, and even outcomes in therapy. For example, P01 noted that “if GPT definitely helps you with journal homework, why not?”, reflecting a practical trust in GenAI’s assistance with therapeutic homework. Participants recognized that when clients felt more comfortable or organized in sharing their experiences (with a bit of GenAI help), it could strengthen the therapy process rather than weakening it.

Several participants gave examples of how client-driven GenAI use had positive ripple effects in therapy. P06, for example, described a case where a client sought advice from ChatGPT after an argument with his roommate. ChatGPT suggested the client should apologize to the roommate, providing direct feedback that the client might have resisted if it came from a human psychotherapist.

*If I challenged a client and said in this scenario, it seems like maybe you need to apologize to your roommate, right? And even if that is true, because the client has done something that is not great, the client may be very mad at me. [However], if [Gen]AI has to offer this idea of challenge, as the client, where does that anger go? It’s just a computer, right? It doesn’t have emotion. And so I think maybe [Gen]AI could deliver more direct and challenging feedback to a client without risk of a therapeutic rupture than a person.*

[P06]

Here, our participants saw GenAI as capable of delivering challenging feedback without risking a rupture in the therapeutic relationship. The client could accept the tough suggestion (to apologize) because it came from an impersonal source, thus preserving the client’s openness with the psychotherapist. Importantly, participants described GenAI not as a substitute for therapeutic challenge or confrontation, but as a relational intermediary that could support client reflection and progress while keeping the therapeutic relationship intact. In P06’s view, GenAI might occasionally say “what needs to be said” in a way that a human psychotherapist strategically might avoid to protect the client’s feelings. Importantly, P06 noted this did not reduce the client’s trust in their human psychotherapist; in fact, it helped the client make progress (repairing a relationship) while avoiding potential defensiveness toward the psychotherapist.

Another psychotherapist, P13, recounted how a client’s use of ChatGPT became a bridge in the therapy session rather than a barrier. The client had chatted with ChatGPT about their symptoms and feelings before the appointment. P13 illustrated how clients’ usage of GenAI could be a topic to start the conversations naturally, boosting client confidence and comfort to share their thoughts during sessions:

*[ChatGPT] allows me to ask questions, like, “Oh, what did you [two] talk about?*” *or “How did it arrive at that conclusion?*” *They’ll answer, “I started saying I’m having these symptoms — X, Y, Z...*” *ChatGPT helped them [clients] organize their thoughts and gave us a starting point. I think it made people feel more comfortable and confident... talking about their experience.*

[P13]

In this scenario, the GenAI functioned as a conversation rehearsal or journaling tool, priming the client to share more openly in session. By externalizing some of the client’s initial thoughts, ChatGPT lowered the barrier to disclosure, making it easier for the client to articulate experiences face-to-face with P13. The psychotherapist observed that this preparatory step reduced the client’s anxiety and increased their willingness to explore their feelings in depth. Crucially, P13 did not feel supplanted by the GenAI; the psychotherapist’s role remained central in interpreting, contextualizing, and building on what emerged from the client-GenAI interaction. This finding reflects how most participants viewed such GenAI usage as a supplementary aid that could facilitate communication and self-expression while preserving and potentially enhancing the core psychotherapist-client relationship.

#### Subtheme 2: Distrust Arises When GenAI Threatens Authentic Human Connection

##### Overview

While psychotherapists were positive about GenAI as a tool, their trust shifted to distrust when considering scenarios where GenAI might encroach on the therapeutic relationship itself. Participants grew skeptical and uneasy about GenAI in therapy whenever it seemed to mimic human empathy, form bonds with clients, or otherwise interfere with the formation of real human relationships. These speculative scenarios triggered strong resistance. Psychotherapists emphasized that effective psychotherapy depended on genuine human connection—often referred to as therapeutic alliance or bond—which involved authentic trust, empathy, and reciprocal emotional engagement. They identified GenAI’s limitations in emotional authenticity, responsiveness, and embodied presence as serious barriers to maintaining this bond. In other words, even though GenAI can imitate empathetic language, it cannot provide the true interpersonal experience of being with another caring human. As discussed below, psychotherapists worried that clients forming illusory relationships with GenAI could be misleading or even harmful, and that a GenAI’s lack of genuine emotion could undercut the healing power of empathy and trust in therapy.

##### Inauthentic (“Parasocial”) Relationships With GenAI

Several psychotherapists drew analogies between a client chatting with a GenAI and the parasocial relationships people develop with media figures or celebrities. Parasocial relationships are one-sided bonds where a person feels a strong connection or intimacy with someone (often a public figure) who is not actually reciprocating that relationship [41]. P04 compared client-GenAI interactions to parasocial relationships, such as those people form with celebrities, highlighting their superficial and ultimately misleading nature:

*A common example is you feel like you know your favorite celebrity, but you really don’t. But you have an attachment to them. If they pass away, you are extremely sad. You feel that loss, you grieve it. You know, those are parasocial relationships. So developing something like that with your GenAI bot as, in a therapeutic realm, I think, is not appropriate.*

[P04]

P04 highlighted the illusory nature of the bond: the client might feel cared for by the GenAI, but in reality, there is no mutual relationship—the GenAI does not “know” the client or genuinely care, just as a celebrity does not know their grieving fan. P04 feared that a client might invest emotionally in a GenAI, only to be hurt upon realizing that this relationship was a 1-way projection. Such a realization could be emotionally devastating (analogous to grieving a celebrity’s death) and might even undermine the client’s trust in real relationships. The implication is that trusting a GenAI as if it were a person is fundamentally misguided, and psychotherapists felt a duty to discourage such attachments for the client’s well-being. Similarly, participant P14 echoed concerns about the implications of normalizing uncertain relationships between clients and GenAI in therapeutic contexts, noting that:

*I do think part of counseling is the relationship that is formed. And again, what does it mean to have a relationship with a non human and like? Is that something that we want or not? And is that something that should be encouraged or not?*

[P14]

Both P04 and P14 were essentially asking: if clients start treating GenAI bots as psychotherapists or friends, do we risk eroding the value of real human-human therapeutic bonds? They emphasized their distrust toward the idea of GenAI-client “relationships,” viewing them as shallow imitations that could mislead clients and ultimately prove harmful. Their perspective taps into broader debates on whether emotional bonds with anthropomorphized GenAI are healthy or not; indeed, psychologists have noted that while such bonds might feel real, they lack true reciprocity and can risk shaping unrealistic expectations about human relationships.

##### Lack of Genuine Emotional Bond and Empathy

Beyond the concept of parasocial attachment, psychotherapists also expressed concern that interacting with an emotionally simulated agent could stunt clients’ ability or desire to form meaningful human connections. Participants noted that much of therapy’s healing power stemmed from experiencing genuine empathy and coregulation with another person. GenAI, no matter how cleverly it reflected a client’s words, fundamentally lacked human emotion, life experience, and a nervous system—it cannot truly “feel” or share in the client’s pain and joy.

As P11 reflected on this with her experiences in talking with GenAI and feeling real comfort, she questioned, “do we want that to be where we go as a profession, they call it the healing arts.” P11 was grappling with the ambivalence: a GenAI might provide momentary comfort (eg, through kind words at 2 AM when no one else is around), but if therapy were to shift toward GenAI, something essential would be lost. She noted that “therapy is not a science, is not formulaic, but is about people.” Thus, shifting therapy to GenAI would strip away its essence as an art grounded in human connection, not a formula that can be executed. P11 even pondered whether making GenAI more human-like for our own convenience “somehow cheapens our experience of being human, or cheats us out of the chance to have real human connection.” P11’s sentiment indicated a sense of distrust: even if GenAI “works” on the surface to soothe someone, relying on it for emotional support might erode the value of human-to-human experiences.

Such interpersonal neural synchronization is only possible through face-to-face human interaction, involving elements such as eye contact, voice tone, and somatic empathy that a GenAI agent cannot easily reproduce. P9 touched on this when she explained that in the therapy room:

*There are mirror neurons in both of our brains... When I take a deep breath, [the client] subconsciously takes a deep breath. When I model a healthy expression of emotion, their mirror neurons are doing the same… There’s a neurological component of therapy that would be lost with GenAI.*

[P09]

In highlighting this embodied coregulation, P09 was pointing to the subtle yet powerful ways a psychotherapist’s presence helped a client feel safe and understood on a biological level. Participants widely felt that a GenAI—even one programmed to respond empathetically in text—could not replicate these rich, multichannel human signals of empathy. Thus, psychotherapists were unwilling to trust GenAI with the core work of forging an emotional bond.

P10 captured the risk that GenAI’s unconditional supportiveness could create a comfortable bubble that ultimately harms client growth. She noted that “learning to tolerate not feeling good can often be part of psychotherapy.” In real therapy, a good clinician sometimes needs to challenge clients’ perceptions or allow them to sit with discomfort as part of healing. P10 wondered if a “GenAI-generated therapy” would instead become “more of a ‘thumbs up’ scenario, creating a loop of people hearing what they want to hear, and thus feeling good.” If a GenAI companion always praises or agrees with the user (either by design or to avoid upsetting them), this always-supportive feature will give clients an illusion of feeling good temporarily and avoid difficult, necessary conversations with real people. Over time, this feedback loop might reduce tolerance for the messiness of real human relationships, and clients could stall in their therapeutic progress. As P10 put it, clients might “cut off real human connections” and bypass the harder work of connecting with others and facing real-world challenges if they grow too comfortable with a GenAI that never challenges them. Such an outcome would be the opposite of what therapy strives for, which is to help clients function better in their lives and relationships outside therapy.

### Subtheme 3: From Personal Fear to Systemic Skepticism—Commercial Incentives Stoke Distrust

Another major theme in psychotherapists’ discussions of trust was the tension between seeing GenAI as a helpful partner in therapy versus viewing it as a potential competitor or replacement. Such tension affected their willingness to trust technology. When GenAI was framed as augmenting their work—handling ancillary tasks, extending care between sessions, or providing additional resources—psychotherapists were generally open-minded and even optimistic. In those cases, GenAI aligned with their professional values, and they could trust it to support better outcomes. However, when the conversation turned to GenAI possibly replacing human psychotherapists, participants expressed strong distrust, often accompanied by anxiety and even a sense of threat to their professional identity.

We found that psychotherapists’ fears evolved from that initial shock toward more pragmatic skepticism. They started scrutinizing real-world trends that might lead to GenAI-driven replacement of psychotherapists. One major factor was the way some technology companies are promoting mental health GenAI products. Participants noted that a number of startups have begun rolling out “therapy bots” or GenAI coach apps. Officially, these are often marketed as assistive tools, but psychotherapists suspected the subtext was to test the waters for full replacement. P13, for instance, mentioned seeing posts on LinkedIn about new chatbot services:

*They’re [mental health chatbot startups] trying to say, “Oh, this is going to be an assistant,*” *but you can tell by the way they’re working it that they’re almost trying to say, hey, we can just do this therapy bot... put in all the information... and it’s gonna replace therapy instead of having to talk to a therapist.*

[P13]

The design and messaging of these products made P13 feel that the endgame for some developers is to bypass human psychotherapists altogether. This sense was reinforced by other participants (P11, P17) who observed how GenAI mental health platforms often come with bold claims or marketing hype. P17 warned that a slickly marketed GenAI could “scam potential clients into thinking they can get [the same] help” from a bot, when in reality that was not advised. Similarly, P11 worried that for-profit GenAI platforms might make “false promises” that lure people into viewing GenAI as nearly equivalent to a human psychotherapist. These comments suggest that psychotherapists’ distrust of GenAI was not only about GenAI’s capabilities, but also about the intentions and honesty of those deploying it. If GenAI is oversold as a reliable replacement for therapy, psychotherapists fear the public could be misled and that clients might forgo seeking real help, with potential harm.

Beyond the companies pushing GenAI, participants highlighted structural pressures that could drive GenAI-as-psychotherapist scenarios. A concern was the role of insurance and health care payers. In many health care systems, including mental health care, decisions about what services are covered (and at what rate) profoundly influence practice. Psychotherapists suspected that if GenAI therapy is seen as significantly cheaper and more efficient, insurers might encourage or even force a shift in that direction. P10 cynically remarked that “[Gen]AI methodology is going to be promoted by insurances [as] a cheaper, better, quicker fix.” P13 similarly noted the temptation for efficiency:

*From an insurance company side—you can be like, “Oh, well, a chatbot can talk to a thousand people a day.*” *That sounds great for efficiency.*

[P13]

Psychotherapists worried that they and their clients could be placed in a no-win situation, where financial incentives pressure everyone toward GenAI solutions regardless of actual therapeutic value. The mere possibility of this happening fueled a lot of distrust and even resentment toward GenAI in our interviews. It is not that these psychotherapists believe a GenAI could truly do the job as well as they can. In fact, they asserted it cannot, for all the human reasons discussed earlier, but the threat is that decisions outside their control (by companies or insurers) might force the issue. Whenever GenAI was envisioned as a substitute for a human psychotherapist, participants’ trust evaporated. They viewed that narrative with distrust and, at times, with a sense of having to justify their own worth in the face of automation.

In summary, psychotherapists in our study navigated a delicate balance of trust and distrust toward GenAI in therapeutic settings. When GenAI supported them and their clients—acting as a friendly assistant that respects the primacy of human connection—they largely trusted it and saw promise. However, when GenAI appeared to usurp human roles or relationships, they responded with firm distrust, rooted in emotional intuition and professional reasoning. These findings highlight the importance of context and role definition in shaping trust: as the literature on automation suggests [21,42], people trust technology only when its use aligns with their goals and values and withdraw trust if it threatens or violates their goals. For psychotherapy, the inviolable goal is to promote genuine human healing and connection. GenAI’s role in this domain, according to our participants, will depend on whether it is designed to honor and enhance, rather than redefine or replace, the human-centric nature of therapeutic relationships.

Across these findings, we see that psychotherapists’ trust and distrust in GenAI are fundamentally about professional role identity. The introduction of GenAI into therapeutic contexts surfaces latent questions about what it means to be a psychotherapist, such as what tasks are core to the role, what knowledge and judgment are uniquely human, and where the boundaries of professional responsibility lie. When participants resisted GenAI involvement in case conceptualization, expressed discomfort with clients forming “relationships” with chatbots, or worried about insurance-driven replacement, they were not merely assessing GenAI’s technical capabilities; they were articulating and defending the boundaries of their professional role. Such dynamics can be understood through role theory [42]. GenAI’s entry into therapy creates potential role ambiguity (uncertainty about which tasks belong to the clinician vs the technology), role conflict (tension between efficiency pressures and relational values), and opportunities for role clarification (explicit articulation of what remains uniquely human). Participants’ insistence on control, their task-specific boundaries, and their concerns about parasocial client-AI relationships all reflect efforts to resolve these tensions in favor of preserving the clinician’s interpretive and relational primacy.

In this sense, GenAI serves as a clarifying lens: by confronting what GenAI can and cannot do, psychotherapists articulate more explicitly what they believe only a human clinician can provide—authentic empathy, embodied presence, ethical accountability, and the capacity to work through relational ruptures. These articulations contribute to ongoing professional discourse about the nature of psychotherapeutic expertise in an increasingly technological landscape.

## Discussion

### Principal Results

Our findings reveal that psychotherapists primarily used GenAI for low-risk, supportive tasks (eg, notes, brainstorming, and homework), while drawing firm boundaries around high-stakes clinical reasoning (eg, case formulation and diagnosis) and any opaque handling of client information. As a result, psychotherapists’ trust in GenAI is highly context-dependent and rooted in maintaining human control and relationships. Finally, we concluded our findings as: psychotherapists’ control as the locus of trust based on their current GenAI adoption, the management of the psychotherapist-client-GenAI triad to protect the therapeutic alliance, and the systemic pressures and fears of exclusion that shape implications for future policy and design.

First, our findings show that trust hinges on professional autonomy (eg, the “hand-on-the-wheel” rule) and concrete controls (eg, prompt scoping, editability, and data deletion) that allow psychotherapists to supervise what goes in and what comes out. Even when the same GenAI tool was technically capable of other functions, psychotherapists were reluctant to shift trust to unfamiliar uses outside their control. For psychotherapists, even a slight shift in control, or a perceived loss of it, can substantially reduce the psychotherapists’ confidence. They integrate GenAI into their workflow only on their own terms, within the boundaries of their professional knowledge and practice.

Psychotherapists’ insistence on concrete controls (eg, scoping GenAI prompts, editing GenAI-generated content, and ensuring data privacy) echoes findings in other professions that emphasize oversight in using GenAI [43-45]. For example, in domains such as finance and accounting, professionals also exhibit conditional trust in GenAI and demand human oversight to meet privacy, compliance, and accuracy requirements [46]. Indeed, many accounting firms (including large firms in the Big Four) have introduced GenAI to assist with tasks such as auditing and tax preparation while keeping a human auditor in the loop [47]. However, psychotherapists practice an even stricter form of conditional trust. Given the extraordinary sensitivity of therapeutic work, where mistakes can carry deeply personal or even harmful consequences for clients, trust in automation is “squeezed”—carefully rationed and contextually negotiated. Consistent with prior research on trust in high-risk settings [48,49], our participants’ trust was conservative by necessity: they only trusted GenAI when its use aligned with their goals, values, and ethical duties, and they withdrew trust immediately if the GenAI’s role threatened those principles.

However, maintaining “control” through careful prompting and boundary-setting does not necessarily guarantee actual control over how GenAI shapes interpretation. As algorithmic systems operate within preexisting biases and conversational frames, even skilled users may be subject to subtle influences that confirm rather than challenge their interpretive directions. The distinction between actual and perceived control is clinically important as a psychotherapist who believes they are steering the GenAI may, in fact, be receiving outputs shaped by the model’s training patterns in ways that reinforce existing assumptions. Such a gap between perceived and actual control interacts with professional role identity. Psychotherapists’ expertise traditionally includes recognizing when their own biases may be influencing clinical judgment—a form of reflective practice. GenAI complicates this by introducing an interlocutor whose biases are opaque and whose “agreement” may feel like validation. Future research should examine whether and how clinicians can develop literacy about these dynamics to engage with GenAI more reflexively.

Taken together, our findings reveal that psychotherapists’ conditions for trust extend beyond retaining veto power or technical oversight. Trust is conditional on maintaining professional role identity—not merely having a “hand on the wheel,” but preserving the clinician’s interpretive authority, ethical responsibility, and relational primacy. When GenAI threatens to shift these role boundaries, even if the clinician technically retains control, trust erodes. In relationally intensive professions such as psychotherapy, “human-in-the-loop” is necessary but not sufficient; the human must remain in the loop as a clinician with a defined professional role, not merely as a monitor or editor of outputs. Such a role-identity framing could help explain why participants resisted GenAI for case conceptualization (a task central to clinical identity) while accepting it for documentation (a task peripheral to their core professional self-concept).

Second, our findings add nuance to understanding the relationship among psychotherapists, clients, and GenAI by examining counselors’ observations of (and hopes for) client-led use of GenAI in therapy. Participants generally supported GenAI as a supplementary aid for clients, but only when it complemented, not competed with, the human relationship. Trust was maintained when a client’s use of GenAI enriched the therapy process under the psychotherapist’s guidance. For example, some clients had experimented with GenAI tools for personal journaling, mood tracking, or skills practice between sessions, and psychotherapists viewed these as positive and adjunctive activities as long as they could review and integrate the GenAI-generated content into treatment. To participants, the GenAI tool functioned as a bridge—facilitating communication and self-reflection—while the core psychotherapist-client bond was preserved or even strengthened. These scenarios illustrate how GenAI can serve as a therapeutic supplement that empowers clients (by providing on-demand exercises or feedback) without undermining the clinician’s authority or the authenticity of the human connection.

While psychotherapists expressed openness to client-led uses of GenAI, as reflected in remarks such as “why not,” participants stressed that acceptance and these benefits only hold under specific conditions, when clients are actively engaged in therapy, and the clinician can oversee the GenAI’s role. Unsupervised or excessive reliance on GenAI by clients raised ethical red flags for many practitioners. In psychotherapists’ view, a GenAI that provided easy answers or constant validation might become an emotional crutch, potentially impeding clients’ growth by sidestepping the discomfort and vulnerability through which therapeutic change occurs. Several psychotherapists noted the risk of clients developing an illusory relationship with a chatbot that mimics empathy but lacks genuine human understanding. They voiced concern that unvetted advice or faux-empathy from GenAI could misguide clients or undercut the healing power of authentic human empathy in therapy. Thus, even the same tools that help organize thoughts between sessions were seen as double-edged: without guardrails, those tools might shape clients’ thinking in ways the psychotherapist cannot monitor or correct. This highlights the importance of embedding GenAI use within ongoing professional support, rather than allowing it to operate as a parallel “psychotherapist.”

Third, systemic forces, such as insurance reimbursement models, health care policies, and technology marketing, further complicate the psychotherapist-client-GenAI triad. Our findings reveal that psychotherapists’ fear of being “replaced” by GenAI stems less from its raw capabilities than from institutional pressures. Many participants expressed concern that decisions by insurers, employers, or health care systems might push GenAI solutions into therapy for the sake of cost or efficiency, sidelining human clinicians against their will. Indeed, signs of these systemic shifts are already visible. In the United States, Medicare announced that as of January 2025, it will reimburse providers for prescribing certain US Food and Drug Administration–cleared digital mental health therapeutics [50]. Private insurers such as Aetna and Cigna have also begun partnering with digital mental health platforms and covering app-based therapeutic services. These developments reflect a growing institutional embrace of GenAI-driven and digital tools to expand access and reduce costs. However, psychotherapists in our study saw a potential dark side: exclusion from the care loop if stakeholders treat GenAI as a cheap substitute for human therapy. They worried about a “no-win situation” where financial incentives pressure clinics to replace portions of therapy with automated services, regardless of client benefit. Such a possibility fueled a great deal of distrust and even resentment toward GenAI among participants. Crucially, it was not that psychotherapists believed a GenAI could do their job just as well—on the contrary, they were confident in the unique value of human care—but rather that decisions outside their control might force the issue (eg, an insurance company mandating a chatbot as a first line of support). Whenever GenAI was imagined as a substitute for a human psychotherapist, participants’ tentative trust evaporated, replaced by staunch distrust and a need to justify their professional worth.

Psychotherapists also noted mixed signals in the policy landscape that mirror this tension. On one hand, policymakers are cautiously enabling GenAI integration in mental health care through reimbursement and innovation incentives. On the other hand, there is growing recognition of the ethical and quality concerns: for example, some US jurisdictions are moving to outright ban certain uses of AI in psychotherapy. In August 2025, the state of Illinois enacted a law prohibiting the use of AI to provide psychotherapy or make clinical decisions [51], while still allowing AI for “administrative and supplementary support” under a provider’s oversight. This law was explicitly framed as protecting patients from unregulated AI or GenAI tools and preserving the jobs of human psychotherapists. Such contradictory responses—from enthusiastic adoption to legal restriction—highlight a core societal debate: Should GenAI be embraced to improve mental health accessibility and efficiency, or curtailed to preserve the human core of therapy? Our participants overwhelmingly rejected either extreme position. The psychotherapists’ stance was one of measured, principled integration: GenAI should be used only in ways that support—and never replace or diminish—the therapeutic relationship. Psychotherapists in our study are not “Luddites” resisting technology out of fear; instead, they are concerned practitioners seeking to ensure GenAI is implemented responsibly and relationally. Their distrust is not a reflexive fear of job loss per se, but a deeper worry about clients being left without meaningful human connection if economic structures push GenAI-only solutions as a shortcut. In fact, many envisioned constructive roles for GenAI (some called it a potential “glue” in therapy) that could enhance rapport, support client progress, and deepen engagement, but only if these tools are introduced thoughtfully, with ethical guardrails and human oversight at every step.

### Limitations and Future Implications

A limitation of this study is that our interview participants may represent a group of psychotherapists with greater initial interest in GenAI than the average practitioner, as they voluntarily opted in through our recruitment emails to complete the interviews. Their perspectives may therefore be more exploratory or engaged compared with those of clinicians who chose not to participate. Our participants were also US-based and demographically skewed (predominantly White, with a relatively larger proportion of women). We did not systematically examine whether perspectives varied across demographic characteristics (eg, gender, race or ethnicity, and years of practice). As a result, the trust and distrust dynamics reported here may not fully reflect the diversity of clinician perspectives and may transfer differently across countries, health care systems, and regulatory contexts. Additionally, we did not assess participants’ primary therapeutic orientation (eg, cognitive-behavioral, psychodynamic, humanistic, and integrative). In the US context, some licensed psychotherapists practice eclectically or identify with integrative approaches, but orientation may nonetheless shape attitudes toward structured tools and the conceptualization of the therapeutic relationship. For example, practitioners with a strong humanistic or relational orientation may be more skeptical of GenAI’s capacity to support authentic connection, while those with cognitive-behavioral orientations may be more receptive to GenAI-assisted psychoeducation or homework. Future research should explicitly examine whether psychotherapists from different schools (eg, the psychodynamic, humanistic, and cognitive-behavioral) differ in their trust calibration and in the conditions under which they would accept GenAI involvement in therapy. Finally, the rapid pace of GenAI development means that our findings reflect psychotherapists’ perceptions at a particular moment in time. As GenAI tools continue to evolve in capability, regulation, and clinical integration, psychotherapists’ trust, concerns, and usage practices may shift in ways not fully captured here.

Despite these limitations, the findings remain highly relevant for clinicians and designers seeking to understand how psychotherapists use GenAI in real clinical contexts, how they negotiate trust and distrust under different conditions, and how they navigate clients’ independent use of GenAI. The themes identified in this study can guide design considerations, inform professional training needs, and highlight the types of support clinicians require when confronted with GenAI in real-world practice.

Future GenAI tools for psychotherapy should be designed to enhance clinician oversight and preserve clinical judgment, rather than shifting interpretive authority to the system. Our findings suggest “minimum conditions” for trust and adoption, including clinician-facing controls to review outputs before use, edit or annotate content to reflect clinical nuance, and delete materials that are inaccurate or clinically unhelpful. Trust also depends on transparency that helps clinicians understand what the model is responding to (eg, visible inputs and omissions) and recognize overconfident or overly generic guidance. Thus, clear data governance, such as storage, retention, access, and use for model improvement, is also essential for informed privacy decisions.

In addition, future researchers could also explore how GenAI can strengthen communication between psychotherapists and clients. Participants described how “GenAI in the loop” could support communication by offering direct or challenging prompts, helping clients organize thoughts, or assisting them in articulating emotions more clearly. Developers may consider GenAI features that act as a bridge between clients and psychotherapists—for example, translating clinical terminology into laypeople’s language or generating structured summaries that help psychotherapists quickly understand clients’ concerns. Such features could enhance clarity and mutual understanding while preserving the psychotherapist’s primary role in treatment.

Participants also highlighted opportunities for GenAI-supported psychoeducation and training. As GenAI becomes increasingly common in clients’ daily lives and in clinical workflows, trainees may benefit from working with GenAI early to learn ethical and appropriate use. Interactive GenAI-simulated patients could also provide customizable training scenarios with different patient backgrounds, symptom profiles, or emotional intensities that are not always available in traditional role-plays or standardized-patient exercises. These adaptive environments may help trainees build practical skills and prepare for future clients who already integrate GenAI into their mental health routines.

Furthermore, the tension between systemic pressures and therapeutic values points to future implications for policy and governance. There is a clear need for professional guidelines and regulations that reaffirm the centrality of human psychotherapists in any GenAI-augmented care model. Organizations such as the APA have begun developing guidance to help psychotherapists navigate GenAI in practice, and our findings underscore how crucial such efforts are. Policies must strike a balance so that GenAI can be leveraged to extend care (eg, through approved digital therapeutics) without eroding standards of care or marginalizing clinicians. Psychotherapists may also require training to work effectively alongside GenAI tools and to advocate for their patients’ needs in dialogues with employers or insurers deploying these tools. Equipping psychotherapists with digital literacy and ethical decision-making frameworks for GenAI can help them retain a sense of agency and protect client welfare when new technology is introduced.

### Conclusions

In this work, we conducted an interview study with 18 psychotherapists to explore how psychotherapists perceive, trust (or distrust), and incorporate GenAI into their everyday practice. We found that psychotherapists exhibit a cautious, conditional trust in GenAI: they embrace these tools for administrative and low-risk tasks (eg, note-taking, training simulations, and between-session client exercises) but are wary of using GenAI for core clinical decisions or any role that might infringe upon the therapeutic relationship. By recognizing the conditions under which psychotherapists are willing to trust GenAI, we can inform the development of tools and policies that empower psychotherapists and safeguard clients. Moving forward, it will be crucial to pursue interdisciplinary research and dialogue that continue to balance innovation with empathy, ensuring that future GenAI in mental health strengthens the art of therapy instead of undermining it.

## Supplementary material

10.2196/88932 Multimedia Appendix 1 Interview guide used to explore psychotherapists’ experiences, trust and distrust, and perceived roles of GenAI in practice. GenAI: generative artificial intelligence.

10.2196/88932 Checklist 1 COREQ 32-item checklist.

## Abbreviations

AI: artificial intelligence
APA: American Psychological Association
COREQ: Consolidated Criteria for Reporting Qualitative Research
GenAI: generative artificial intelligence
