# Supplementary material for: Psychotherapists’ Trust, Distrust, and Generative AI Practices in Psychotherapy: Qualitative Study
Source: J Med Internet Res. 2026 Apr 2;28:e88932. doi: 10.2196/88932 (PMC13051054; doi:10.2196/88932)
Supplement: Multimedia Appendix 1 [file jmir-v28-e88932-s001.docx]

**Semistructured Interview Guide**

**Brief introduction of the research study:** The goal of this interview is to understand psychotherapists’ practices, attitudes, and trust regarding the use of GenAI in psychotherapy.

**Consent form:** Ask participants to sign the consent form before the interview.

**Introduction to the interview:**

- **Let’s start with the interview.** I will ask you a set of questions to get to know you better. I have a list of questions here that I will be reading from. There are no right answers and no wrong answers. The goal is to gain an understanding of your experiences and perspectives.
- We may report the results of this project in publications and presentations. Any information that you share will not be linked to you in any way. Your name and any other identifying information will never be attached to the information shared.
- **Do you have any questions?**
- **Audio Recording:** I would like to audio record this session to make sure we capture everything you say today. Is it OK for me to audio record?
- **Compensation:** within 1-2 weeks;

**Semistructured Interview Guide**

Before each interview, we provided participants with a shared definition of *trust* and *distrust*, framing trust as a willingness to rely on GenAI, and distrust as an active withholding of reliance, to ensure conceptual clarity throughout the study.

Experiences:

1. Could you briefly describe your current practices (e.g., client population, therapeutic approach)?
2. How have you used GenAI in your work? Can you share your experiences or provide some examples? ***Note: If participants did not have any GenAI experiences, skip to question (3).***
   1. It seems that you’ve had a [positive/negative] experience with GenAI. Could you elaborate more? What factors contributed to this perception?
   2. Have these experiences raised any concerns about GenAI in therapeutic practice?
   3. Are you still using GenAI in your therapeutic practice after these experiences?
      1. *If yes*, what aspects of your past experiences have encouraged you to continue? How do those effects influence your continuous engagement?
      2. *If not, was your decision influenced by these experiences? Or are there any other reasons? Why?*
   4. *How do your experiences influence your trust and distrust towards GenAI in mental health*
3. We have a document from the American Psychological Association outlining GenAI’s integration into psychotherapy. After you review this document, we will have several questions to ask:
   1. Are you familiar with it? If so, what were your initial impressions?
   2. The document outlines possible applications of GenAI in psychotherapy. What are your thoughts on these potential uses?
   3. The document discusses ethical considerations related to GenAI in psychotherapy. Do you agree with the concerns mentioned? Are there any additional ethical concerns you think should be addressed?
   4. What specific concerns do you have about incorporating GenAI into psychotherapeutic practice? Are these concerns related to your particular clinical setting, your therapeutic approach, or other factors?
   5. Do you use GenAI in general? If so, what’s your experience? How do you feel about those experiences? Will those experiences lead you to consider using GenAI in psychotherapy in the future or not?
4. Could you describe any tasks or tools your clients have mentioned? ***Note: If psychotherapists did not report their clients’ independent use of GenAI in mental health in the screening survey, skip to question (5).***
   1. Do you have concerns about their usage? Are they general or specific to certain clients (e.g., demographic groups)?
   2. How does client GenAI use compare with therapist GenAI use? (e.g., benefits, risks, ethics)
   3. Has this changed your own views or practices regarding GenAI in therapy?
   4. Has seeing clients use GenAI affected your trust or distrust? How?
5. Please consider a hypothetical scenario: There has been a growing trend of clients using GenAI or generative GenAI chatbots independently.
   1. What do you think about this trend?
   2. What potential benefits or risks do you foresee for clients who engage with GenAI tools on their own?
   3. How does the hypothetical possibility of clients’ usage of GenAI in mental health influence your trust and distrust?
6. What factors make you more likely to trust a GenAI tool?
7. What factors make you more likely to distrust a GenAI tool?
8. How do you envision the relationship among psychotherapists, clients, and GenAI going forward?
9. How do your perceived relationships influence your trust and distrust?
