# Supplementary material for: Psychotherapists’ Trust, Distrust, and Generative AI Practices in Psychotherapy: Qualitative Study
Source: J Med Internet Res. 2026 Apr 2;28:e88932. doi: 10.2196/88932 (PMC13051054; doi:10.2196/88932)
Supplement: Checklist 1 [file jmir-v28-e88932-s002.pdf]

Consolidated criteria for reporting qualitative studies (COREQ): 32-item checklist

No	Item	Guide questions/description	Notes
**Domain 1: Research team and reflexivity**			
The research team<br>The multidisciplinary research team had expertise in qualitative study (JK, YZ, AP), GenAI (JK, YZ), and psychotherapy (AP, YZ)			
**Personal Characteristics**			
1	Interviewer/facilitator	Which author/s conducted the interview or focus group?	JK and YZ conducted the semi-structured interviews.
2	Credentials	What were the researcher's credentials? E.g. PhD, MD	JK : BS,<br>YZ: PhD,<br>AP: PhD
3	Occupation	What was their occupation at the time of the study?	JK: Research Assistant<br>YZ: Assistant Professor in Computer Science<br>AP: Assistant Professor in Counselor Education
4	Gender	Was the researcher male or female?	All the researchers are female
5	Experience and training	What experience or training did the researcher have?	All the researchers had experience with qualitative methods, including conducting interviews
**Relationship with participants**			
6	Relationship established	Was a relationship established prior to study commencement?	The research team did not have any contact with participants before obtaining informed consent. Researchers had no professional or ongoing relationship with the participants.
7	Participant knowledge of the interviewer	What did the participants know about the researcher? e.g. personal goals, reasons for doing the research	Participants were aware that this was a research project to explore the experiences of GenAI in psychotherapy and their perceived trust/distrust. The research team explained that the research goal was to understand their experiences, perceptions, and perceived GenAI's role in their therapeutic relationship.

8	Interviewer characteristics	What characteristics were reported about the interviewer/facilitator? e.g. Bias, assumptions, reasons and interests in the research topic	The research team had an interest in psychotherapists' current use of GenAI in psychotherapy and how they negotiate their trust and distrust regarding the use of GenAI.
**Domain 2: study design**			
**Theoretical framework**			
9	Methodological orientation and Theory	What methodological orientation was stated to underpin the study? e.g. grounded theory, discourse analysis, ethnography, phenomenology, content analysis	The research team used the General Inductive Approach.
**Participant selection**			
10	Sampling	How were participants selected? e.g. purposive, convenience, consecutive, snowball	Participants were recruited through professional mailing lists, social media, and snowball sampling.
11	Method of approach	How were participants approached? e.g. face-to-face, telephone, mail, email	The research team approached participants via email.
12	Sample size	How many participants were in the study?	There were 18 participants in the study.
13	Non-participation	How many people refused to participate or dropped out? Reasons?	Two eligible individuals scheduled interviews but did not attend (no-shows). No follow-up information was available regarding reasons for non-attendance
**Setting**			
14	Setting of data collection	Where was the data collected? e.g. home, clinic, workplace	The semi-structured interview sessions were conducted via Zoom.
15	Presence of non-participants	Was anyone else present besides the participants and researchers?	There were no people present during the data collection besides participants and researchers.
16	Description of sample	What are the important characteristics of the sample? e.g. demographic data, date	The mean working experiences of participants is 9.7 years and 14 of 18 participants are women.
**Data collection**			
17	Interview guide	Were questions, prompts, guides provided by the authors? Was it pilot tested?	See Interview Study Procedures Page 6

18	Repeat interviews	Were repeat interviews carried out? If yes, how many?	There were no repeat interviews with the same participants.
19	Audio/visual recording	Did the research use audio or visual recording to collect the data?	All interviews were audio recorded with the permission of the participants.
20	Field notes	Were field notes made during and/or after the interview or focus group?	Researchers made field notes during the interviews.
21	Duration	What was the duration of the interviews or focus group?	The individual interviews with psychotherapists had an average duration of 60 minutes.
22	Data saturation	Was data saturation discussed?	Data saturation was reached on all major topics.
23	Transcripts returned	Were transcripts returned to participants for comment and/or correction?	Transcripts were not returned to participants for comment and/or correction.
**Domain 3: analysis and findings**			
**Data analysis**			
24	Number of data coders	How many data coders coded the data?	The lead author conducted line-by-line coding, assigning low-level descriptive codes to relevant text segments. Two additional members of the research team independently coded overlapping subsets of transcripts using the evolving codebook and met with the lead author in weekly analytic meetings to compare interpretations and resolve discrepancies through consensus. In total, three coders contributed to the coding process.
25	Description of the coding tree	Did authors provide a description of the coding tree?	Data Analysis. Researchers described the coding tree in detail from low-level to high level.
26	Derivation of themes	Were themes identified in advance or derived from the data?	Themes were derived from the data in an inductive manner.
27	Software	What software, if applicable, was used to manage the data?	MAXQDA24 was used during the coding of interview data.
28	Participant checking	Did participants provide feedback on the findings?	There was no member check performed by the research team.

Reporting			
29	Quotations presented	Were participant quotations presented to illustrate the themes / findings? Was each quotation identified? e.g. participant number	Key findings of this study were supported with selected quotes in text.
30	Data and findings consistent	Was there consistency between the data presented and the findings?	All findings were derived from the data and all themes are supported by illustrative quotes.
31	Clarity of major themes	Were major themes clearly presented in the findings?	Results. Major themes were derived from the data and are clearly defined by a section or subsection title.
32	Clarity of minor themes	Is there a description of diverse cases or discussion of minor themes?	Results. Researchers described different cases and used representative quotes.
